# Pragmatic Trials for Noncommunicable Diseases: Relieving Constraints

**DOI:** 10.1371/journal.pmed.1001986

**Published:** 2016-03-29

**Authors:** Anushka Patel, Ruth Webster

**Affiliations:** The George Institute for Global Health, University of Sydney, Sydney, Australia

## Abstract

In this month's editorial, *PLOS Medicine* Editorial Board member Anushka Patel and Ruth Webster discuss how applying rules for drug efficacy trials can impede pragmatic trials of interventions for noncommunicable diseases.

At the 65th World Health Assembly in 2012, all World Health Organization member states made a historic commitment to reduce premature deaths from noncommunicable diseases (NCDs, including cardiovascular diseases, diabetes, cancer, and chronic respiratory diseases) by 25% by 2025. Subsequently, the World Health Assembly in 2013 agreed to adopt a global monitoring framework that included nine ambitious global NCD targets for 2025 [1]. These targets address key risk factors including tobacco and alcohol use, physical inactivity, high salt intake, high blood pressure, diabetes, and obesity and also the availability of basic technologies and medicines for the prevention and treatment of major NCDs.

Many of these targets reflect the fact that a wide range of safe and effective treatments for the prevention and management of many NCDs already exists. These treatments have been the product of decades of “discovery research” utilising high-quality, large-scale randomised controlled trials to produce reliable evidence of safety and efficacy. Unfortunately, translation of these important discoveries has been less than optimal globally, but particularly so in low- and middle-income countries that are now bearing the brunt of the global NCD burden. This translation gap is fuelled by uncertainty about how best to implement effective treatments tested under ideal conditions into the complex and often resource-constrained systems in which they need to be delivered.

Clear demonstration of such knowledge translation gaps has raised the profile of implementation research in NCD prevention and control [2,3]. Implementation research, which has been defined by some as “the scientific inquiry into questions concerning implementation—the act of carrying an intention into effect, which in health research can be policies, programmes, or individual practices,” can be used to address a range of relevant questions and is therefore characterised by a broad range of research methodologies [4]. One method is the pragmatic randomised controlled trial (pRCT), important to answer questions about whether there is a high probability that a particular intervention will produce an expected outcome in a particular context. Unlike “discovery” research, which is primarily designed to determine the safety and efficacy of an intervention under ideal conditions, pRCTs aim to reflect effectiveness of strategies to utilise such interventions under “real world” conditions. The outcomes that pRCTs generate are likely to be particularly relevant for local implementation solutions but can also result in substantial generalisable knowledge.

However, a number of challenges exist in designing and conducting useful pRCTs, particularly in the context of the requirement to balance the needs for internal and external validity, as well as in protecting the interests of research participants. Other challenges reflect the fact that "usual practice" occurs within the context of complex adaptive systems (e.g., an institution or a health system), not subject to the linear pathway of causality often assumed for efficacy trials. For pRCTs, researchers need to consider introducing some design features that are important for contextual understanding but not appropriate for classic RCTs that generate data on safety and efficacy. Similarly, those who evaluate research proposals (e.g., peer-review panels of funding agencies, ethics committees, and regulatory bodies) should carefully consider the context and purpose of a pragmatic study and avoid imposing unwarranted conditions that could undermine its potential value. Examples of problematic design features include the following:

1. Strict inclusion and exclusion criteria (other than appropriate criteria based on known safety and efficacy data) that limit generalisability.
2. Implementation of data collection or other trial processes in such a way that substantially alters the study intervention or usual care.
3. Individual consent procedures (where there are no or minimal additional risks compared to usual care) that will likely impede use of an intervention because of the influence of consent procedures on work flow.
4. Specifying “unusual” usual care, namely care which does not reflect typical practice in the environment in which the intervention would be applied.
5. Restriction of the purchase of goods and services by research participants, particularly where the costs of goods or services are likely to influence uptake in the “real world” context.
6. Strict standardisation of the study intervention to the exclusion of variations that are to be expected following implementation within complex adaptive systems. Context is important in implementation research and can vary substantially between and within health systems and over time. Such variation does not negate the value of investigating effectiveness of the intervention in a pRCT, as long as variations in implementation are captured and adequately described.
7. Attempting to introduce blinding where this is inherently not possible. While lack of blinding can introduce bias particularly in the context of subjective patient-reported outcomes, knowledge of receipt (or lack of receipt) of an intervention may influence behaviour and adoption in “real life” implementation. Capturing such information (e.g., through process evaluations embedded within a pRCT) may be extremely valuable.
8. Inadequate emphasis on outcomes beyond establishing effectiveness that relate to service delivery such as adoption, acceptability, feasibility, fidelity, cost, coverage, and sustainability. Also important might be the measurement of potential unintended consequences of implementing a new intervention within a complex health system.

Examples of some of these challenges can be found in a range of research studies that aimed to understand the potential role of fixed-dose combination drugs (“polypills”) in reducing the treatment gaps among people at high risk of cardiovascular disease, in whom safety and efficacy of component medications is established, with guidelines universally recommending their use. It has been theorised that implementation strategies that utilise polypills with generic components may help reduce treatment gaps by reducing costs and complexity of drug regimens. A pilot trial conducted in Sri Lanka failed to demonstrate benefits of a polypill-based strategy over “standard care” in terms of blood pressure and cholesterol reduction; however, standard care as implemented in this trial was subsequently found to be highly unusual, with much greater use of recommended medications compared to known “real world” practice patterns [5]. To some extent, this may have been driven by changes to standard care introduced only in the context of the trial. In a large pRCT of a polypill-based strategy in the UK and India, the polypill was provided for free (as this was defined as the “study drug”), while usual care participants may not have received similar subsidies [6]. If cost of treatment is a real impediment to appropriate drug use, this difference may lead to inaccurate assumptions about “real world” effectiveness.

These issues are not all easily addressed, but more awareness, consideration, and debate are required to maximise the value of pRCTs, which represent one important type of implementation research. Careful consideration of these issues is important not only for researchers developing and conducting pRCTs but also for those responsible for approving proposed research. Clinical trial activity is usually assessed within ethics and regulatory frameworks that are primarily designed for efficacy studies of new drugs and devices. A regulatory burden that is disproportionate to the typical level of risk in pRCTs evaluating implementation of treatments of known safety and efficacy can seriously undermine the usefulness of such research. For NCDs, pRCTs are important to identify the best way to implement the plethora of available effective preventive interventions. If current constraints are not addressed, meeting many of the 2025 voluntary goals for NCD control may be even more challenging.

## Abbreviations

NCD: noncommunicable disease
pRCT: pragmatic randomised controlled trial
